# Mesenchymal circulating tumor cells and Ki67: their mutual correlation and prognostic implications in hepatocellular carcinoma

**DOI:** 10.1186/s12885-023-10503-3

**Published:** 2023-01-05

**Authors:** Xihua Yang, Hanghang Ni, Zhan Lu, Jie Zhang, Qian Zhang, Shangwu Ning, Lunan Qi, Bangde Xiang

**Affiliations:** 1 Department of Hepatobiliary Surgery, Guangxi Medical University Cancer Hospital, 71# Hedi Road, Qingxiu District, Nanning, Guangxi 530021 People’s Republic of China; 2 Department of Surgical Oncology, Chenzhou No. 1 People’s Hospital, Chenzhou, People’s Republic of China; 3 Key Laboratory of Early Prevention and Treatment for Regional High-Frequency Tumors, Ministry of Education, Nanning, People’s Republic of China; 4 Guangxi Liver Cancer Diagnosis and Treatment Engineering and Technology Research Center, Nanning, People’s Republic of China

**Keywords:** Hepatocellular carcinoma, Circulating tumor cells, Ki67, Epithelial–mesenchymal transition, Prognosis

## Abstract

**Background:**

Mesenchymal circulating tumor cells (M-CTCs) may be related to tumor progression, and Ki67 expression is known to be involved in tumor proliferation. The aim of the present study was to explore the relationship between M-CTCs and Ki67 in hepatocellular carcinoma (HCC) and their ability to predict prognosis.

**Methods:**

Peripheral blood samples were obtained from 105 HCC patients before radical surgery. CTCs were isolated using CanPatrol enrichment and classified via in situ hybridization. Ki67 expression in HCC tissue was assessed through immunohistochemistry. Potential relationships of M-CTC, Ki67 with clinicopathological factors and prognosis were evaluated. Overall survival (OS) was analyzed using the Kaplan–Meier method and Cox regression. The prognostic efficacy of M-CTC, Ki67 and both together (M-CTC + Ki67) was assessed in terms of time-dependent receiver operating characteristic (ROC) curves and Harrell's concordance index.

**Results:**

Of the 105 patients, 50 were positive for M-CTCs (count ≥ 1 per 5 mL) and 39 showed high Ki67 expression (≥ 50% tumor cells were Ki67-positive). The presence of M-CTC was significantly associated with alpha-fetoprotein (AFP) ≥ 400 ng/mL (*P* = 0.007), tumor size ≥ 5 cm (*P* = 0.023), multiple tumors (*P* < 0.001), poorly differentiated tumors (*P* = 0.003), incomplete tumor capsule (*P* < 0.001), Barcelona Clinic liver cancer (BCLC) stage B or C (*P* < 0.001), microvascular invasion (MVI) (*P* = 0.05) and portal vein tumor thrombosis (PVTT) (*P* = 0.006). High Ki67 expression correlated with AFP ≥ 400 ng/mL (*P* = 0.015), tumor size ≥ 5 cm (*P* = 0.012), incomplete tumor capsule (*P* < 0.001), MVI (*P* = 0.001), PVTT (*P* = 0.003), advanced BCLC stage (*P* = 0.01), and vessel carcinoma embolus (VCE) (*P* = 0.001). M-CTC positively correlated with Ki67. Patients positive for M-CTCs had a significantly shorter OS than patients negative for them. Similarly, high Ki67 expression was associated with a significantly lower OS. The high-risk group (positive for M-CTCs and high Ki67 expression) had worse OS than the other groups (*P* < 0.0001). Uni- and multivariate analyses showed that OS was independently predicted by M-CTC [hazard ratio (HR) 1.115; *P* < 0.001], Ki67 (HR 1.666; *P* = 0.046) and the combination of both (HR 2.885; *P* = 0.008). Based on ROC curves and the concordance index, the combination of M-CTC and Ki67 was superior to either parameter alone for predicting the OS of HCC patients.

**Conclusions:**

The presence of M-CTC correlates with high Ki67 expression in HCC patients, and both factors are associated with poor prognosis. Furthermore, the combination of M-CTC and Ki67 is a useful prognostic indicator for predicting OS in patients with HCC after hepatectomy, performing better than either parameter on its own.

**Supplementary Information:**

The online version contains supplementary material available at 10.1186/s12885-023-10503-3.

## Background

Hepatocellular carcinoma (HCC) is one of the most frequent malignant neoplasms, ranking among the top five leading causes of cancer-related deaths worldwide [1, 2]. Surgical resection remains the primary potentially curative treatment, yet long-term survival remains unsatisfactory, mainly due to a high incidence of recurrence and mortality. Currently, there is no reliable method for predicting the fate of individual patients with HCC [3]. Serum alpha-fetoprotein (AFP), although it is considered the most reliable biomarker of HCC diagnosis, performs poorly as a prognostic indicator. For example, 40–60% of HCC patients exhibit normal AFP levels [4], making it unreliable as a marker for monitoring recurrence after resection. Other prognostic factors for HCC have been proposed, including the tumor / node / metastases (TNM) staging system and the Barcelona Clinic Liver Cancer (BCLC) staging system; however, the accuracy of these systems can vary substantially [5]. Thus, a sensitive and effective biomarker is urgently required to help predict patients’ outcomes, which may improve patient management and therefore survival.

One such biomarker may be circulating tumor cells (CTCs), which have potent proliferative and metastatic abilities. CTCs may shed from the primary tumor into the bloodstream before or even during surgical resection, then travel throughout the body via the systemic circulation. Detecting CTCs by liquid biopsy may substantially improve prognosis prediction and detection of recurrence [6, 7]. CTCs can be classified into three subpopulations: epithelial (E-CTCs), mesenchymal (M-CTCs), or an intermediate subtype undergoing the epithelial-to-mesenchymal transition (E/M-CTCs) [8]. These subtypes can be distinguished based on expression of surface markers. The epithelial-to-mesenchymal transition (EMT), a phenotypic change marked by the loss of epithelial characteristics and the acquisition of invasive mesenchymal properties, is considered essential for metastasis [9]. CTCs gain mesenchymal features via EMT and drive HCC metastasis [10, 11]. Levels of M-CTC not only represent the progression and state of the disease, but also serve as a prognostic marker. In fact, recent studies have demonstrated that M-CTCs are better at predicting prognosis than the total number of CTCs in patients with lung [12], gastric [13], or breast cancer [14].

Accurately predicting the prognosis of HCC patients will likely require taking into account other factors in addition to M-CTCs. One such factor may be cell proliferation, as assessed based on expression of the DNA-binding nuclear protein Ki67. This protein is expressed in all phases of mitosis except the G0 phase, and it consistently shows high sensitivity and specificity as a marker of proliferative cells [15]. Ki67 upregulation may help drive the proliferation of malignant tumor cells, making it a potential biomarker of tumor aggressiveness and poor prognosis [16].

Combining the characteristics of tumor proliferation and CTCs may lead to more accurate prediction of prognosis of patients with various solid neoplasms, including non-small cell lung cancer [17] and renal cell carcinoma [18]. However, we are unaware of reports assessing the relationship between M-CTC and Ki67 in patients with HCC. Therefore, the present study aimed to investigate whether the M-CTC count in peripheral blood and Ki67 expression in tumor tissues correlate with each other and whether they can, alone or together, predict survival of patients with HCC.

## Methods

### Study population

A total of 105 patients were enrolled between March 2014 and May 2017 at the Guangxi Medical University Cancer Hospital (Nanning, China). The inclusion criteria were as follows: (1) definitive HCC diagnosis based on World Health Organization criteria [19]; (2) liver function in Child–Pugh A stage and Performance Status Test score of 0–1; (3) treatment by R0 resection, defined as complete macroscopic removal of the tumor, negative resection margin and no detectable residual intra- or extrahepatic metastatic lesions; (4) no history of anticancer treatment, such as trans-arterial chemoembolization or targeted therapy; and (5) availability of complete medical records. Patients with other systemic tumors and autoimmune diseases were excluded. Peripheral venous punctures within one week before surgery were used to analyze preoperative blood markers, including AFP and hepatitis B virus DNA (HBV-DNA). Cut-off values were 400 ng/mL for AFP and 5.0 × 10^2^ IU/mL for HBV-DNA, as recommended by the assay manufacturer.

The study was conducted according to the principles of the Declaration of Helsinki and was approved by the Ethics Review Committee of the Affiliated Cancer Hospital of Guangxi Medical University (approval number: LW2022121). The requirement for informed consent to participate was waived by the Ethics Review Committee of the Affiliated Cancer Hospital of Guangxi Medical University. The reason for the waiver of informed consent is because all patients, on admission, consented for their anonymized medical data to be analyzed and published for research purposes.

## *CTC isolation and* in situ *hybridization*

At 1–2 days before resection, peripheral blood (5 mL) was collected into anticoagulant-coated tubes for assays of CTCs. CTCs were isolated using the CanPatrol enrichment method [20, 21]. Briefly, erythrocytes were removed using red blood cell lysis buffer (Sur Exam, Guangzhou, China), and the remaining cells were resuspended for 5 min in 4% formaldehyde dissolved in phosphate-buffered saline (PBS; Sigma, St. Louis, MO, USA). Next, the blood was filtered using a filter tube (Sur Exam) fitted with a membrane containing pores of diameter 8 µm (Sur Exam) on a E-Z96 vacuum manifold (Omega, Norcross, GA, USA) attached to a vacuum pump (Auto Science, Tianjin, China) set to 0.08 MPa [22].

In situ hybridization was used to detect mRNAs expressing the epithelial biomarkers (EpCAM and CK8/18/19), as well as the mesenchymal biomarkers (Vimentin and Twist). Assays were performed in 24-well plates (Corning, NY, USA). Cells on the membrane were treated with a protease (Qiagen, Hilden, Germany), then subjected to in situ hybridization (see Supplementary Table S1 for probe sequences) as previously described [20, 23]. In our study, P53 gene R249S mutation was detected in the DNA of both primary tumors and CTCS, but not in non-tumor liver tissues, thus confirming that CTCs originate from primary tumors [20]. A total CTC count or M-CTC count of 0 per 5 mL was defined as negative; ≥ 1 per 5 mL, as positive.

### Ki67 immunohistochemistry

Samples from representative areas of HCC tumors excised during surgery were fixed with formalin, embedded in paraffin, deparaffinized with xylene, incubated for 2 min in repair solution under high pressure at high temperature, allowed to cool to room temperature, and finally washed three times with PBS for 15 min. Samples were then treated with 3% H_2_O_2_ for 10 min, washed with PBS as above, incubated with primary antibody against Ki67 (1:100; Gene Tech, Shanghai, China), and washed again with PBS as above. Samples were incubated for 120 min at 37 °C using horseradish peroxidase-labelled secondary antibody. Diaminobenzidine (DAB) was used as the chromogen. Samples were washed with water for 10 min, stained with hematoxylin, then washed until colorless. Ki67-positive staining was defined as the presence of brownish-yellow granules in the nucleus. The proportion of positively stained cells was calculated as the Ki67 proliferative index (Figure S1). Samples were assessed independently by two pathologists who were blind to clinical and follow-up data.

### Patient follow-up

The 105 patients were followed up every 1–2 months for the first year and every 3 months thereafter, with the last follow-up on July 31, 2021. Follow-up procedures included ultrasonography, dynamic computed tomography, magnetic resonance imaging, and serum AFP measurement. Recurrence was diagnosed in patients who (1) showed elevated AFP as well as evidence of recurrence based on contrast-enhanced ultrasonography, computed tomography or magnetic resonance; or (2) showed normal AFP but evidence of recurrence based on two of the three imaging modalities; or (3) whose biopsies of new lesions showed pathology indicative of recurrence [24]. Recurrence location was classified as intra- or extrahepatic. OS was defined as the time from surgery until all-cause death or until the last recorded follow-up visit.

### Statistical analysis

Statistical analyses were performed using SPSS 26.0 (IBM, Chicago, IL, USA). The graphs were drawn using GraphPad Prism version 8.0 (GraphPad Software, La Jolla, CA, USA) and R version 4.1.2 (http://www.r-project.org/). All statistical assessments were two-tailed, and results were considered significant if associated with *P* < 0.05.

Patient characteristics were analyzed using descriptive statistics. Intergroup differences were assessed for significance using the Pearson chi-squared test or Fisher’s exact test. The optimal cut-off value for defining Ki67 positivity was determined by X-tile (version 3.6.1) [25]. Logistic regression was performed to estimate odds ratios (ORs) and corresponding 95% confidence intervals (CIs) in order to identify associations among clinical features, M-CTC count and Ki67 expression. Correlation between M-CTC and Ki67 was assessed with Spearman’s correlation coefficient.

Kaplan–Meier survival curves were generated, then compared using the log-rank test. Cox proportional hazard regression was used for univariate and multivariate analyses to identify prognostic factors associated with OS. When appropriate, hazard ratios (HRs) were reported together with 95% confidence intervals (CIs). The area under the receiver operating characteristic curve (AUC) was measured to evaluate the ability of total CTC, M-CTC, Ki67, or the combination of M-CTC and Ki67 to predict OS. The time-dependent ROC and concordance index were analyzed using the R packages “timeROC” and “Hmisc”.

## Results

### Patient characteristics

The study analyzed 90 men and 15 women, and the median age of all patients was 46.2 (range, 20–72) years. Most patients (75, 71.4%) had HBV-DNA levels ≥ 5.0 × 10^2^ IU/mL, and just over half (59, 56.2%) had AFP levels ≥ 400 ng/mL. Just under half (42, 40.0%) had poorly differentiated tumors, 82 (78.1%) had primary tumors ≥ 5 cm, 23 (21.9%) had primary tumors < 5 cm, and 40 (38.1%) had multiple tumors. Three of patients were BCLC stage 0, 54 BCLC A, 25 BCLC B and 23 BCLC C. A smaller proportion (41, 39.0%) had an incomplete tumor capsule. Patients’ demographic and clinical characteristics are summarized in Table 1.

**Table 1:** Demographic and clinical characteristics of patients (*N* = 105)

Characteristic	Category	n (%)
Sex	Male	90 (85.7)
	Female	15 (14.3)
Age (years)	< 45	43 (41.0)
	≥ 45	62 (59.0)
HBsAg	Negative	12 (11.4)
	Positive	93 (88.6)
HBV-DNA (IU/mL)	< 5.0 × 10^2^	30 (28.6)
	≥ 5.0 × 10^2^	75 (71.4)
AFP (ng/mL)	< 400	46 (43.8)
	≥ 400	59 (56.2)
Tumor size (cm)	< 5	23 (21.9)
	≥ 5	82 (78.1)
Tumor number	Single	65 (61.9)
	Multiple	40 (38.1)
Edmondson grade	I–II	63 (60.0)
	III–IV	42 (40.0)
Tumor capsule	Incomplete	41 (39.0)
	Complete	64 (61.0)
MVI	Negative	33 (31.4)
	Positive	72 (68.6)
PVTT	Negative	82 (78.1)
	Positive	23 (21.9)
Liver cirrhosis	Negative	4 (3.8)
	Positive	101 (96.2)
BCLC stage	0-A	57 (54.3)
	B-C	48 (45.7)
VCE	Negative	41 (39.0)
	Positive	64 (61.0)

*AFP* alpha-fetoprotein, *BCLC* Barcelona Clinic Liver Cancer, *HBsAg* hepatitis B surface antigen, *HBV-DNA* hepatitis B virus DNA, *MVI* microvascular invasion, *PVTT* portal vein tumor thrombosis, *VCE* vessel carcinoma embolus

### Relative proportions of CTC phenotypes

**Fig. 1:**
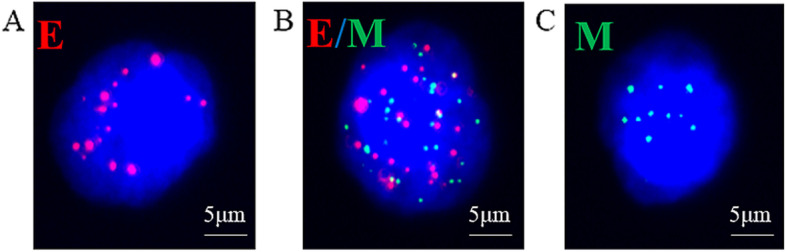
Representative micrographs of circulating tumor cell (CTC) subpopulations. CTCs were stained for epithelial markers (EpCAM and CK8/18/19, red fluorescence) and mesenchymal markers (Vimentin and Twist, green fluorescence). **A** Epithelial CTCs (E-CTCs). **B** Epithelial/mesenchymal hybrid CTCs (E/M-CTCs). **C** Mesenchymal CTCs (M-CTCs). Magnification, 100 ×

**Fig. 2:**
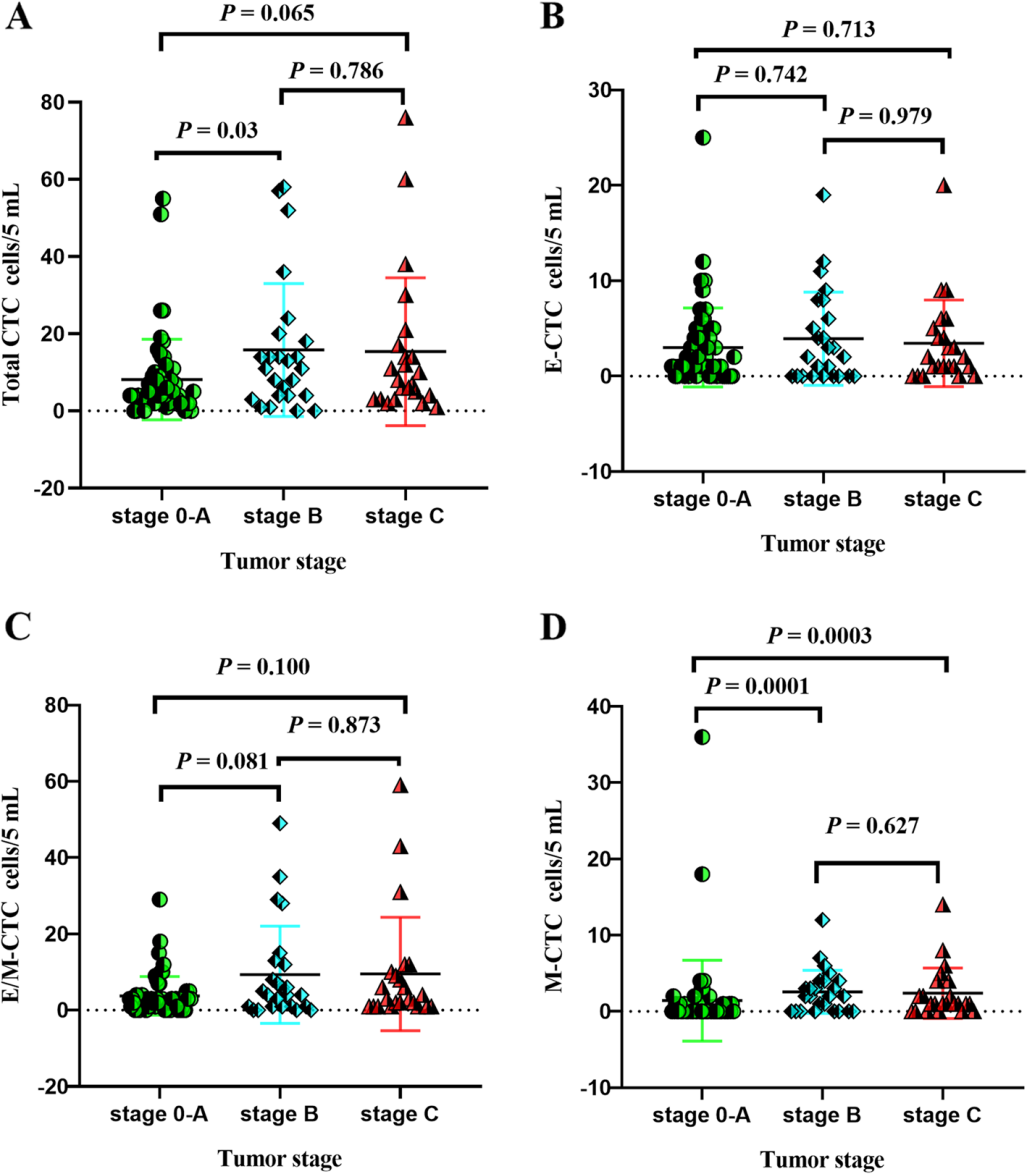
Total circulating tumor cell (CTC) counts and counts for the three CTC subpopulations in hepatocellular carcinoma patients stratified by Barcelona Clinic Liver Cancer (BCLC) stage. **A** Total CTC count. **B** Epithelial CTC (E-CTC) count. **C** Epithelial/mesenchymal hybrid CTC (E/M-CTC) count. **D** Mesenchymal CTC (M-CTC) count

Representative images of the three subtypes of CTCs that were isolated from the 105 HCC patients before surgery are shown in Fig. 1. The overall rate of CTC positivity was 93.3% (range, 0–76%), and the median and mean counts were 6 and 11.55 per 5 mL, respectively (Table 2). Total CTC count tended to increase with disease progression but did not differ significantly between patients in BCLC stage B or C (Fig. 2A). In contrast, E-CTC and E/M-CTC count did not vary with HCC progression (Fig. 2B-C). Notably, the rates of M-CTC positivity were 28% in stages 0-A, 68% in stage B and 78.2% in stage C (Table 2), and the M-CTC count increased with disease progression (Fig. 2D). Therefore, HCC progression in our sample was associated with an increase in the number of CTCs undergoing the EMT. Furthermore, total CTC and M-CTC predicted OS with respective AUCs of 0.554 and 0.614 (Fig. S2). In other words, M-CTC outperformed total CTC for predicting survival.

**Table 2:** Rates of positivity and counts of total CTCs and different CTC subpopulations in HCC patients

BCLC stage	n	n (%) of patients positive for CTCs				Median CTC count (per 5 mL)	Mean CTC count (per 5 mL)	Range of CTC count (per 5 mL)
		Total CTCs	E- CTCs	E/M-CTCs	M-CTCs			
0-A	57	52 (91.2)	43 (75.4)	10 (17.5)	16 (28.0)	5	8.158	0–55
B	25	23 (92.0)	16 (64.0)	21 (84.0)	17 (68.0)	11	15.8	0–58
C	23	23 (100.0)	18 (78.2)	23 (100.0)	18 (78.2)	8	15.35	1–76
total	105	98 (93.3)	77 (73.3)	91 (86.6)	50 (47.6)	6	11.55	0–76

*BCLC* Barcelona Clinic Liver Cancer, *CTCs* circulating tumor cells, *E-CTCs* epithelial CTCs, *M-CTCs* mesenchymal CTCs, *E/M-CTCs* epithelial/mesenchymal hybrid CTCs, *HCC* hepatocellular carcinoma

### Optimal cut-off for classifying samples based on Ki67 expression

Based on immunohistochemistry, an optimal cut-off value was determined using the X-Tile statistical package to classify patients into those showing low or high Ki67 expression. Low expression was defined as tumor tissue in which < 50% of tumor cells were positive for Ki67, while high expression was defined as tissue in which at least half of tumor cells were positive (Fig. S3).

### Association of Ki67 expression with CTC type

**Fig. 3:**
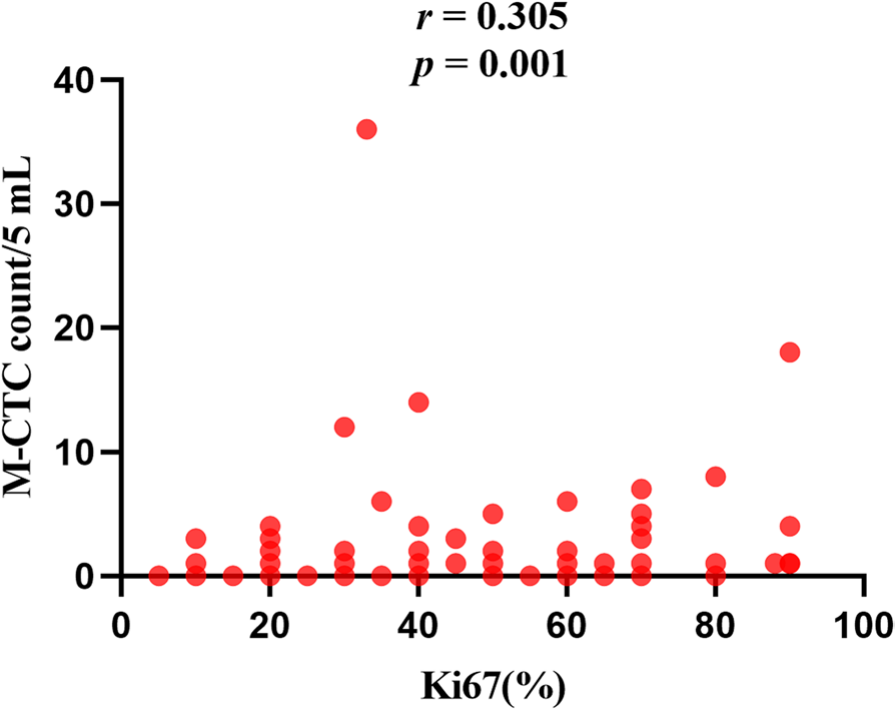
Relationship between preoperative mesenchymal circulating tumor cell count (M-CTC) in peripheral blood and Ki-67 in tumor tissue of HCC patients

Relationships between Ki67 expression and CTCs are shown in Table 3. Patients with high Ki67 expression showed a significantly higher rate of M-CTC positivity than patients with low Ki67 expression (61.5%, 24/39 vs. 39.3%, 26/66; *P* = 0.03). M-CTC showed a weak positive correlation with Ki67 based on Spearman’s correlation coefficient (*r* = 0.305; *P* = 0.001; Fig. 3). In contrast, Ki67 expression was not significantly associated with the rates of positivity for total CTCs (count ≥ 6 per 5 mL), E-CTCs or E/M-CTCs (*P* > 0.05).

**Table 3:** Associations between CTC levels and Ki67 expression

Level	Ki67 expression, n		OR (95% CI)	*P*
	High (≥ 50%)	Low (< 50%)		
Total CTC (per 5 mL)				
Low (˂ 6)	14	34	1	
High (≥ 6)	25	32	1.897 (0.841–4.278)	0.123
E-CTC				
Negative	11	17	1	
Positive	28	49	0.883 (0.363–2.149)	0.784
E/M-CTC				
Negative	3	11	1	
Positive	36	55	2.400 (0.626–9.202)	0.202
M-CTC				
Negative	15	40	1	
Positive	24	26	2.462 (1.092–5.546)	0.03

*CI* confidence interval, *CTC* circulating tumor cell, *E-CTC* epithelial CTC, *M-CTC* mesenchymal CTC, *E/M-CTC* epithelial/mesenchymal hybrid CTC, *OR* odds ratio

### Relationships of M-CTC or Ki67 with clinicopathological characteristics of HCC

Relationships between M-CTC and clinical parameters are shown in Table 4. The rate of M-CTC positivity was significantly higher among patients with AFP ≥ 400 ng/mL (*P* = 0.007), tumor size ≥ 5 cm (*P* = 0.023), multiple tumors (*P* < 0.001), poorly differentiated tumors (*P* = 0.003), incomplete tumor capsule (*P* < 0.001), BCLC stage B or C (*P* < 0.001), MVI (*P* = 0.05) or PVTT (*P* = 0.006). In contrast, age, sex, HBsAg, HBV-DNA, and vessel carcinoma embolus (VCE) were not significantly associated with M-CTC.

**Table 4:** Comparison of clinicopathological characteristics between HCC patients positive or negative for M-CTC

Parameter	M-CTC status		OR (95% CI)	*P*
	Negative (*n* = 55)	Positive (*n* = 50)		
Sex				
Male	46 (83.6)	44 (88.0)	1	
Female	9 (16.4)	6 (12.0)	0.697 (0.229–2.120)	0.525
Age (years)				
< 45	21 (38.1)	22 (44.0)	1	
≥ 45	34 (61.9)	28 (56.0)	0.786 (0.361–1.714)	0.545
HBsAg				
Negative	6 (10.9)	6 (12.0)	1	
Positive	49 (89.1)	44 (88.0)	0.898 (0.270–2.989)	0.861
HBV-DNA (IU/mL)				
< 5.0 × 10^2^	17 (30.9)	13 (26.0)	1	
≥ 5.0 × 10^2^	38 (69.1)	37 (74.0)	1.273 (0.543–2.986)	0.578
AFP (ng/mL)				
< 400	31 (56.4)	15 (30.0)	1	
≥ 400	24 (43.6)	35 (70.0)	3.014 (1.346–6.749)	0.007
Tumor size (cm)				
< 5	17 (30.9)	6 (12.0)	1	
≥ 5	38 (69.1)	44 (88.0)	3.281 (1.175–9.161)	0.023
Tumor number				
Single	42 (76.4)	23 (46.0)	1	
Multiple	13 (23.6)	27 (54.0)	1.273 (0.543–2.986)	< 0.001
Edmondson grade				
I–II	39 (70.9)	24 (48.0)	1	
III–IV	16 (29.1)	26 (52.0)	2.422 (1.092–5.372)	0.03
Tumor capsule				
Incomplete	11 (20.0)	30 (60.0)	1	
Complete	44 (80.0)	20 (40.0)	0.167 (0.070–0.398)	< 0.001
MVI				
Negative	22 (40.0)	11(22.0)	1	
Positive	33 (60.0)	39 (78.0)	2.364 (1.001–5.583)	0.05
PVTT				
Negative	49 (89.1)	33 (66.0)	1	
Positive	6 (10.9)	17 (34.0)	4.207 (1.502–11.785)	0.006
BCLC stage				
0-A	41 (74.6)	16 (52.0)	1	
B-C	14 (25.4)	34 (68.0)	3.013 (1.733–5.238)	< 0.001
VCE				
Negative	26 (47.3)	15 (30.0)	1	
Positive	29 (52.7)	35 (70.0)	2.092 (0.936–4.673)	0.072

*AFP* alpha-fetoprotein, *BCLC* Barcelona Clinic Liver Cancer, *CI* confidence interval, *HBsAg* hepatitis B surface antigen, *HBV-DNA* hepatitis B virus DNA, *M-CTC* mesenchymal CTC, *MVI* microvascular invasion, *OR* odds ratio, *PVTT* portal vein tumor thrombosis, *VCE* vessel carcinoma embolus

Ki67 expression and clinicopathological parameters of HCC are presented in Table 5. Among the 105 HCC tissues, 39 (37.1%) were positive for Ki67. High Ki67 expression was significantly associated with AFP ≥ 400 ng/mL (*P* = 0.015), tumor size ≥ 5 cm (*P* = 0.012), incomplete tumor capsule (*P* < 0.001), MVI (*P* = 0.001), PVTT (*P* = 0.003), advanced BCLC stage (*P* = 0.01), and VCE (*P* = 0.001). Other factors were not significantly associated with Ki67, including age, sex, HBsAg, tumor number, Edmondson grade, or liver cirrhosis.

**Table 5:** Comparison of clinicopathological characteristics between HCC patients with low (< 50%) or high (≥ 50%) Ki67 expression

Parameter	Ki67 expression		OR (95% CI)	*P*
	Low (*n* = 66)	High (*n* = 39)		
Sex				
Male	56 (84.8)	34 (87.2)	1	
Female	10 (15.2)	5 (12.8)	0.824 (0.259–2.614)	0.742
Age (years)				
< 45	26 (39.4)	17 (43.6)	1	
≥ 45	40 (60.6)	22 (56.4)	0.841 (0.377–1.877)	0.673
HBsAg				
Negative	7 (10.6)	5 (12.8)	1	
Positive	59 (89.4)	34 (87.2)	0.807 (0.238–2.740)	0.731
HBV-DNA (IU/mL)				
< 5.0 × 10^2^	21 (53.8)	9 (23.1)	1	
≥ 5.0 × 10^2^	45 (68.2)	30 (76.9)	1.556 (0.628–3.854)	0.34
AFP (ng/mL)				
< 400	35 (53.0)	11 (28.2)	1	
≥ 400	31 (47.0)	28 (71.8)	2.874 (1.230–6.714)	0.015
Tumor size (cm)				
< 5	20 (30.3)	3 (7.7)	1	
≥ 5	46 (69.7)	36 (92.3)	5.217 (1.437–18.944)	0.012
Tumor number				
Single	39 (59.1)	26 (66.7)	1	
Multiple	27 (40.9)	13 (33.3)	0.906 (0.598–1.372)	0.640
Edmondson grade				
I–II	44 (66.7)	19 (48.7)	1	
III–IV	22 (33.3)	20 (51.3)	1.968 (0.878–4.409)	0.1
Tumor capsule				
Incomplete	15 (22.7)	26 (66.7)	1	
Complete	51 (77.3)	13 (33.3)	0.147 (0.061–0.355)	< 0.001
MVI				
Negative	29 (43.9)	4 (10.3)	1	
Positive	37 (56.1)	35 (89.7)	6.858 (2.187–21.508)	0.001
PVTT				
Negative	58 (87.9)	24 (61.5)	1	
Positive	8 (12.1)	15 (38.5)	4.531 (1.699–12.087)	0.003
Liver cirrhosis				
Negative	3 (4.5)	1 (2.6)	1	
Positive	63 (95.5)	38 (97.4)	1.810 (0.182–18.025)	0.613
BCLC stage				
0-A	41 (62.1)	16 (41.0)	1	
B-C	25 (37.9)	23 (60.0)	1.934 (1.174–3.185)	0.01
VCE				
Negative	34 (51.5)	7 (17.9)	1	
Positive	32 (48.5)	32 (82.1)	4.857 (1.879–12.555)	0.001

*AFP* alpha-fetoprotein, *BCLC* Barcelona Clinic Liver Cancer, *CI* confidence interval, *HBsAg* hepatitis B surface antigen, *HBV-DNA* hepatitis B virus DNA, *M-CTC* mesenchymal CTC, *MVI* microvascular invasion, *OR* odds ratio, *PVTT* portal vein tumor thrombosis, *VCE* vessel carcinoma embolus

### Prognostic potential of M-CTC and Ki67 in HCC

**Fig. 4:**
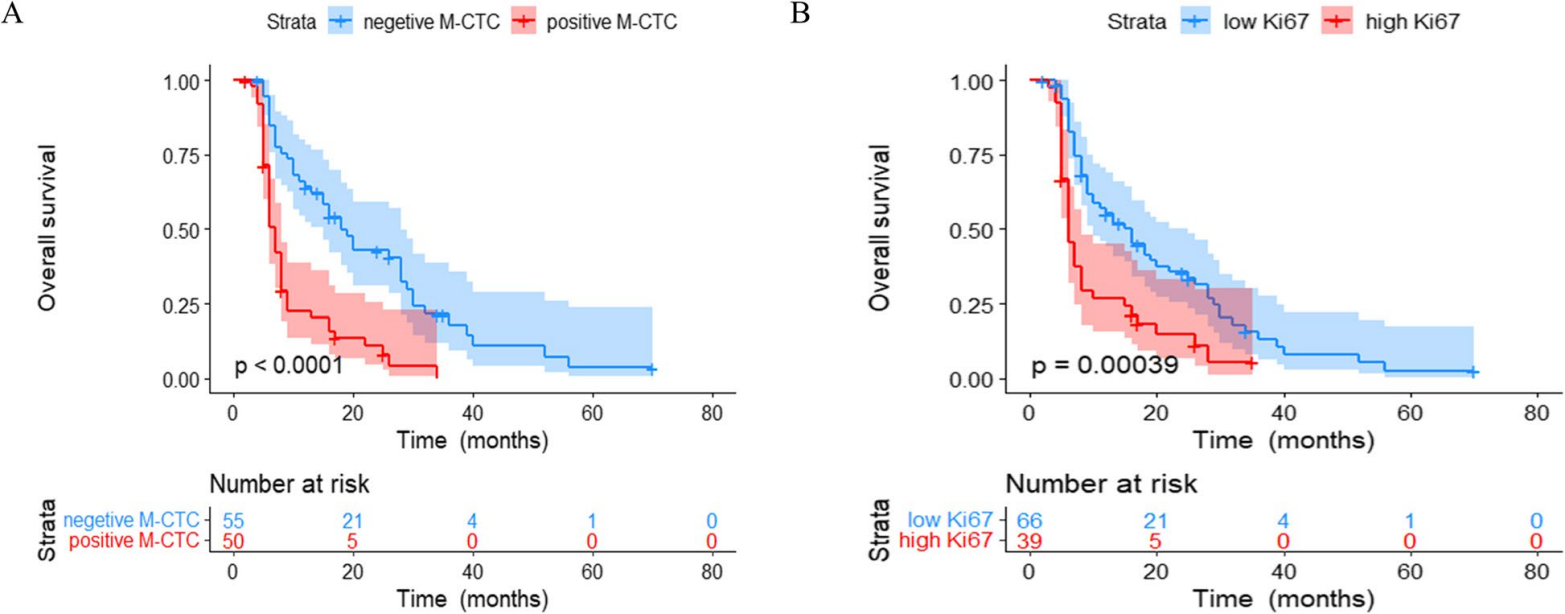
Kaplan–Meier curves for overall survival (OS) based on **A** mesenchymal circulating tumor cell (M-CTC) status (positive or negative) or **B** Ki-67 expression (low or high)

Patients lacking M-CTCs had significantly longer OS than patients positive for M-CTCs (*P* < 0.0001, Fig. 4A). In addition, patients with high Ki67 expression showed worse OS than those with low expression (*P* = 0.00039, Fig. 4B).

To improve the accuracy and stability of prognosis prediction for HCC, we analyzed the M-CTC and Ki67 in combination. In our cohort, patients were classified into three groups: (1) the high-risk group, who were positive for M-CTCs and showed high Ki67 expression; (2) the medium-risk group, who were negative for M-CTCs and showed high Ki67 expression, or were positive for M-CTCs and showed low Ki67 expression; and (3) the low-risk group, who were negative for M-CTCs and showed low Ki67 expression. Our data showed that the combination of M-CTC and Ki67 correlated with AFP, tumor size, tumor number, Edmondson grade, tumor capsule, MVI, PVTT, BCLC stage and VCE (Table 6).

**Table 6:** Correlation of the combination of M-CTC and Ki67 with clinicopathological characteristics of HCC patients

Parameter	Risk group based on the combination of M-CTC and Ki67			*P*
	Low risk	Medium risk	High risk	
	(*n* = 40)	(*n* = 41)	(*n* = 24)	
Sex				
Male	34	34	22	
Female	6	7	2	0.721
Age (years)				
< 45	16	15	12	
≥ 45	24	26	12	0.562
HBsAg				
Negative	4	5	3	
Positive	36	36	21	1.000
HBV-DNA (IU/mL)				
< 5.0 × 10^2^	12	14	4	
≥ 5.0 × 10^2^	28	27	20	0.311
AFP (ng/mL)				
< 400	26	14	6	
≥ 400	14	27	18	0.002
Tumor size (cm)				
< 5	14	9	0	
≥ 5	26	32	24	0.004
Tumor number				
Single	31	23	11	
Multiple	9	18	13	0.025
Edmondson grade				
I–II	27	27	9	
III–IV	13	14	15	0.037
Tumor capsule				
Incomplete	5	16	20	
Complete	35	25	4	< 0.001
MVI				
Negative	19	13	1	
Positive	21	28	23	0.001
PVTT				
Negative	37	33	12	
Positive	3	8	12	< 0.001
Liver cirrhosis				
Negative	3	1	0	
Positive	37	40	24	0.357
BCLC stage				
0-A	31	20	6	
B-C	9	21	18	< 0.001
VCE				
Negative	21	18	2	
Positive	19	23	22	0.001

*AFP* alpha-fetoprotein, *BCLC* Barcelona Clinic Liver Cancer, *HBsAg* hepatitis B surface antigen, *HBV-DNA* hepatitis B virus DNA, *MVI* microvascular invasion, *PVTT* portal vein tumor thrombosis, *VCE* vessel carcinoma embolus

**Fig. 5:**
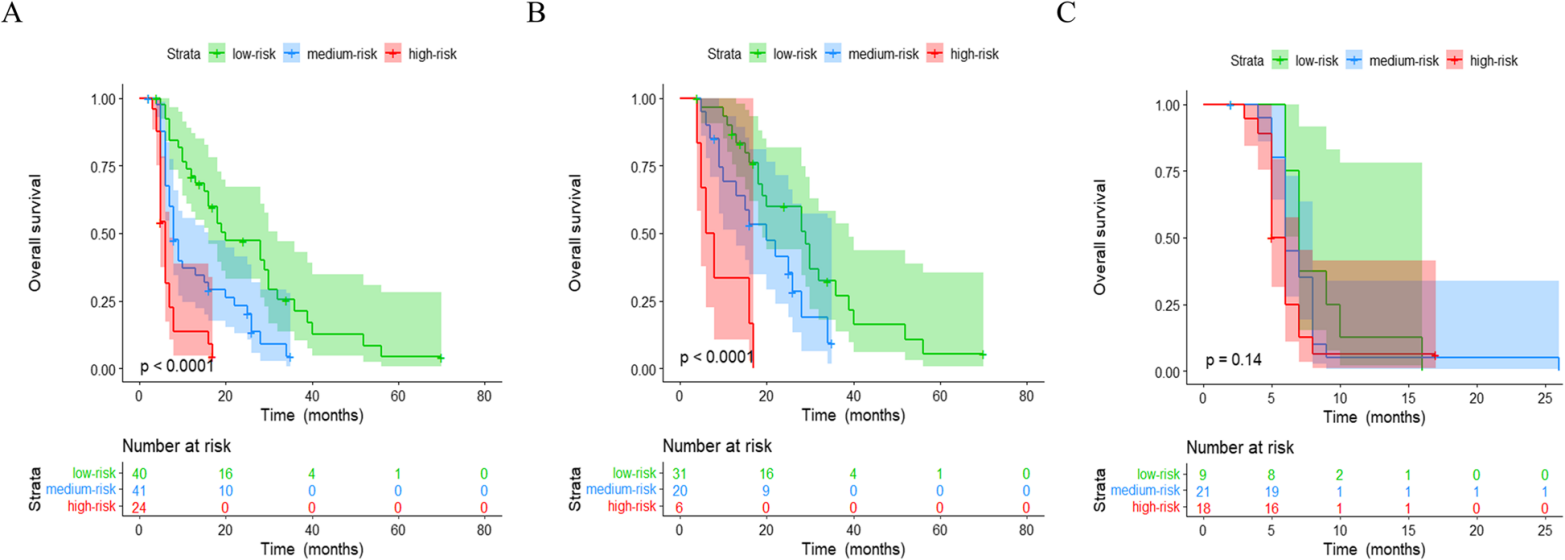
Comparison of overall survival (OS) of patients with HCC stratified by mesenchymal circulating tumor cell (M-CTC) and Ki-67. **A** All patients. **B** Patients in Barcelona Clinic Liver Cancer (BCLC) stages 0-A. **C** Patients in BCLC stages B-C

Kaplan–Meier survival showed that the high-risk group had significantly worse OS than the other two groups (*P* < 0.0001, Fig. 5A). Next, the predictive performance of the combination of M-CTC and Ki67 was assessed for patients in different BCLC stages. Among the 57 patients in BCLC stages 0-A, the high-risk group showed significantly worse OS than the other two groups (*P* < 0.0001, Fig. 5B). However, OS did not differ significantly among patients in the three risk groups who were in BCLC stages B-C (*P* = 0.14, Fig. 5C).

### Univariate and multivariate survival analysis

Univariate analysis of OS identified 12 variables as prognostic factors: AFP, tumor size, tumor number, Edmondson grade, tumor capsule, MVI, PVTT, BCLC stage, VCE, Ki67, M-CTC and the combination of M-CTC and Ki67 (Table 7). Next, two separate multivariate models were generated to avoid multicollinearity among M-CTC, Ki67 and their combination. One model demonstrated that MVI, BCLC stage, Ki67 and M-CTC were independent prognostic factors for OS. The other model revealed that AFP, BCLC stage and the combination of M-CTC and Ki67 had prognostic significance for OS (Table 8).

**Table 7:** Univariate analysis of clinicopathological characteristics associated with overall survival

Parameter	HR	95% CI	*P*
BCLC (B-C)	3.764	(2.659–5.327)	< 0.001
PVTT (positive)	7.380	(3.985–13.664)	< 0.001
Tumor cap (complete)	0.258	(0.163–0.409)	< 0.001
Tumor number (multiple)	1.814	(1.083–3.040)	< 0.001
M-CTC (positive)	3.186	(2.015–5.037)	< 0.001
MVI (positive)	2.768	(1.704–4.496)	< 0.001
AFP (≥ 400 ng/mL)	2.364	(1.499–3.730)	< 0.001
VCE (positive)	2.169	(1.383–3.401)	< 0.001
Ki67 (≥ 50%)	2.251	(1.438–3.525)	< 0.001
Tumor size (≥ 5)	1.814	(1.083–3.040)	0.023
EdmondsIII–IV	1.568	(1.018–2.417)	0.041
Liver cirrhosis (positive)	3.358	(1.041–10.828)	0.042
M-CTC + Ki67	6.025	(3.019–12.024)	< 0.001
HBsAg (positive)	1.696	(0.813–3.539)	0.159
Age (≥ 45)	0.750	(0.485–1.160)	0.196
HBV-DNA (≥ 5 × 10^2^ IU/mL)	1.106	(0.685–1.786)	0.677
Sex (M/F)	0.935	(0.481–1.817)	0.844

*AFP* alpha-fetoprotein, *BCLC* Barcelona Clinic Liver Cancer, *CI* confidence interval, *HBsAg* hepatitis B surface antigen, *HBV-DNA* hepatitis B virus DNA, *HR* hazard ratio, *MVI* microvascular invasion, *M-CTC* mesenchymal circulating tumor cell, *PVTT* portal vein tumor thrombosis, *VCE* vessel carcinoma embolus

**Table 8:** Multivariate analysis of clinicopathological characteristics associated with overall survival

Variable	HR	95% CI	*P*
*Model 1*			
BCLC (B-C)	4.174	(2.402–7.253)	< 0.001
Tumor cap (complete)	0.602	(0.348–1.041)	0.069
M-CTC (positive)	1.115	(1.065–1.167)	< 0.001
MVI (positive)	1.978	(1.179–3.318)	0.010
Ki67 (≥ 50%)	1.666	(1.009–2.749)	0.046
*Model 2*			
M-CTC + Ki67	2.885	(1.317–6.319)	0.008
AFP (≥ 400 ng/mL)	2.014	(1.077–3.764)	0.028
BCLC (B-C)	4.428	(1.881–10.424)	0.001

*AFP* alpha-fetoprotein, *BCLC* Barcelona Clinic Liver Cancer, *CI* confidence interval, *HR* hazard ratio, *MVI* microvascular invasion, *M-CTC* mesenchymal circulating tumor cell

### Prognostic performance of M-CTC, Ki67 and their combination

**Fig. 6:**
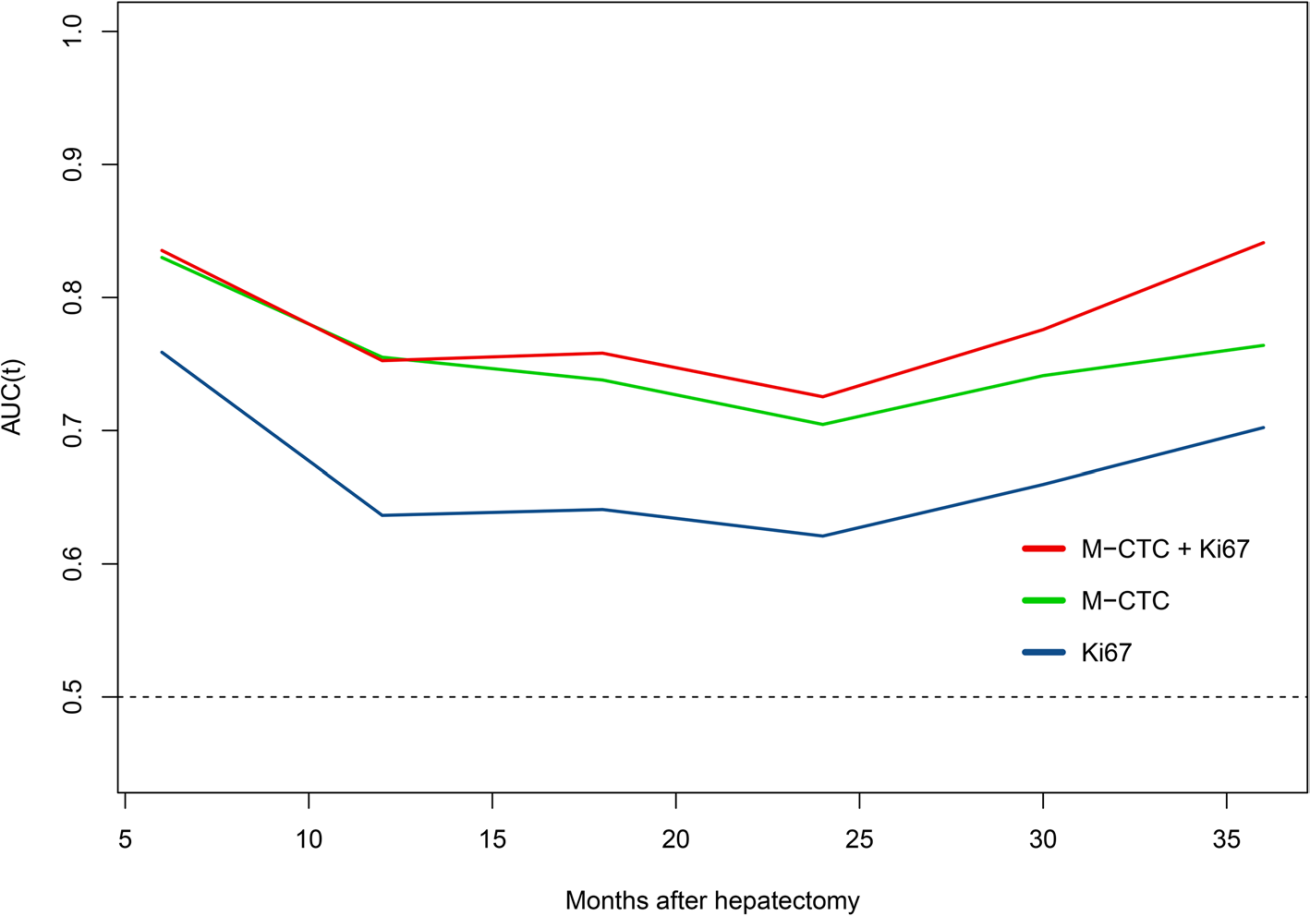
Time-dependent AUC curves of mesenchymal circulating tumor cells (M-CTC), Ki67, and their combination for prediction of overall survival of patients with HCC. Overall survival was predicted at **A** 1 year, **B** 2 years or **C** 3 years after hepatectomy. AUC, area under the curve

**Fig. 7:**
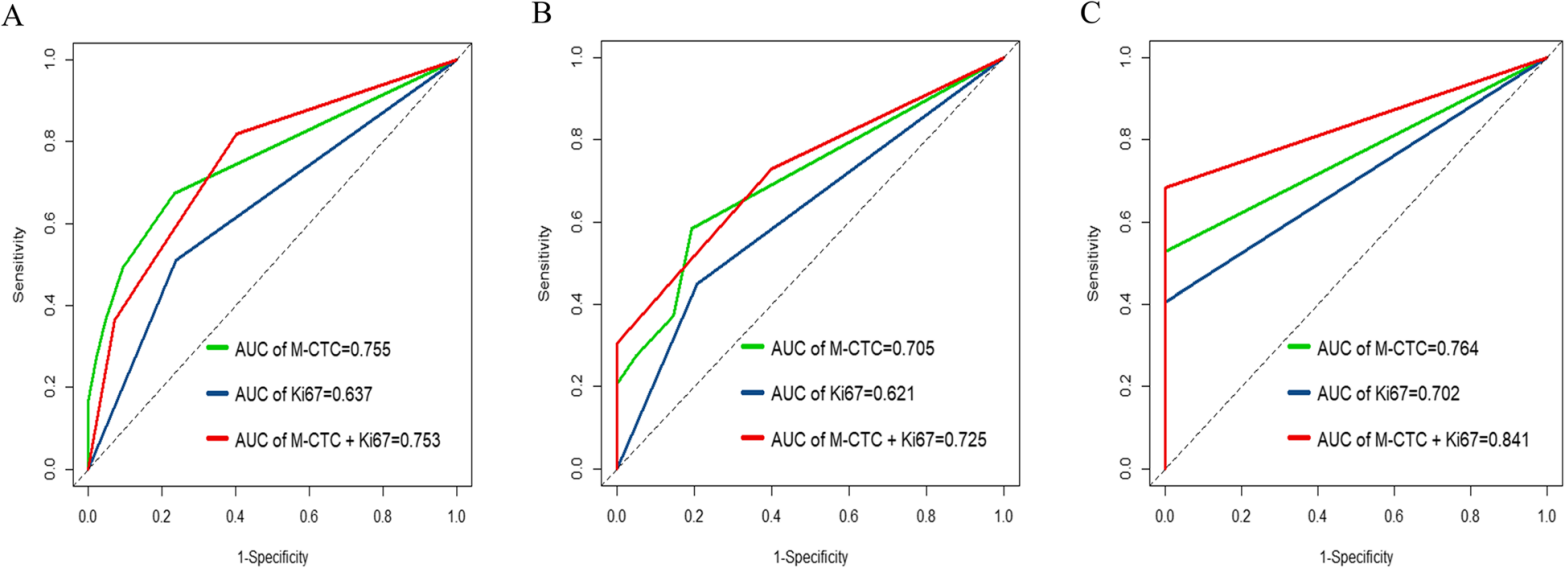
Comparison of time-dependent ROC curves for predicting overall survival of patients with HCC using mesenchymal circulating tumor cells (M-CTC), Ki67, or their combination. AUC, area under the curve. ROC, receiver operating characteristic

Time-dependent ROC curves for predicting OS at 1, 2 and 3 years were generated to compare the performance of M-CTC, Ki67 or their combination. The combination nearly equaled the prognostic power of M-CTC for 1-year OS, and it was superior to the performance of M-CTC or Ki67 for 2- and 3-year OS (Fig. 6). Then we calculated AUC values for M-CTC, Ki67 or their combination. The integration of the estimated AUCs using a time-dependent ROC curve clearly showed that the combination of M-CTC and Ki67 predicted OS better than either parameter alone (Fig. 7). The concordance index for the combination was 0.704, greater than that for M-CTC alone (0.694) or Ki67 alone (0.624).

## Discussion

Improving prognosis for HCC patients requires personalized determination of recurrence risk and careful monitoring after treatment. Here we provide the first evidence that combining M-CTCs and Ki67 may stratify patients according to OS better than either parameter on its own, particularly according to OS at 2–3 years after hepatectomy. Thus, the combination of the two biomarkers may guide treatment and monitoring of HCC patients who have undergone hepatic resection. This combination takes into account essential pathological features, including tumor progression and proliferation. It may become easier to assay the combination as the cost of CTC analysis falls.

CTCs have been validated as a robust biomarker to predict prognosis, so their determination may guide precision treatment of HCC [26]. Assessment of CTCs should include phenotypic identification to characterize cells based on epithelial and mesenchymal markers [27]. M-CTCs are thought to be the most malignant CTCs [20, 28]: several signaling mechanisms protect mesenchymal-like cells from anoikis, including PI3K/AKT, NF-κB, Wnt/β-catenin, and p53/p63 pathways [29]. A high M-CTC percentage is closely associated with expression of CK19 and poor prognosis in HCC [30]. Similar to M-CTCs, expression of Ki67 is strongly related to tumor proliferation and growth.

Mesenchymal tumor cells infiltrate the extracellular matrix by releasing proteolytic degradation enzymes and crossing the basement membrane, they enter the circulation and they eventually extravasate to initiate secondary micro-metastasis [31, 32]. This implies a link between M-CTCs and cancer progression. Indeed, M-CTC count is significantly higher in advanced HCC than in early disease [33], and we found the M-CTC count to predict OS better than the total CTC count. Our study also found that M-CTC positivity was associated with several features of HCC malignancy, including high AFP, larger and multiple tumors, poor tumor differentiation, incomplete tumor capsule, and the presence of MVI and PVTT. This may help explain why patients in our study who were positive for M-CTCs had significantly worse outcomes than those negative for the cells, and why M-CTC was an independent predictor of shorter OS. Previous work has shown that patients positive for M-CTCs are more likely to experience early recurrence [34]. Our previous work revealed that anatomical resection may not be more beneficial than non-anatomical resection for patients positive for M-CTCs [24]. Therefore, M-CTCs should be considered an aggressive subtype, and CTC analysis before surgery can help guide the choice of resection method. We further recommend that HCC patients positive for M-CTCs should be treated with more effective therapies against recurrence and should be monitored closely after treatment.

Our study suggests that in addition to M-CTCs, Ki67 can help individualize treatment and surveillance. Higher Ki67 levels reflect the enhanced proliferative activity of tumor cells. A meta-analysis of 54 studies involving 4,996 HCC patients revealed that Ki67 expression was associated with more advanced tumor stage [35]. However, the tumors in the meta-analyzed studies were dichotomized arbitrarily into those expressing low or high Ki67 values [36–38]. In the present study, we obtained the optimal cut-off value of Ki67 using X-tile software [25], a widely recognized and applied method in many studies. Based on this cut-off, high Ki67 expression correlated significantly with unfavorable clinicopathological features and with significantly worse OS after hepatectomy. In addition, multivariate analysis identified Ki67 as an independent predictor for OS after hepatectomy.

The observed weak link between M-CTC and Ki67 may reflect that CTCs are derived from the primary tumor and therefore share similar characteristics, yet the tumor microenvironment undergoes substantial changes when tumor cells detach from the primary tumor and enter the bloodstream [39]. Our patients classified as high-risk based on positive M-CTC and high Ki67 expression showed significantly shorter OS than those classified as medium- or low-risk, even if their HCC was in early stages. Similarly, prognosis was significantly worse for patients with non-small cell lung cancer who were positive for M-CTCs and had high Ki67 expression than for patients with only one of these two risk factors [17]. We hypothesize that the proliferative effects of Ki67 synergize with the EMT of CTCs to promote HCC progression, ultimately leading to poor prognosis. These results may help improve the risk stratification of patients with HCC. In particular, high-risk patients may benefit from a higher intensity of adjuvant therapy and follow-up.

This study has several limitations. First, it was a single-center retrospective study with a relatively small sample, which increases the risk of selection bias and lack of generalizability to other patient populations. Our findings should be validated and extended in larger, multi-center, prospective studies. Second, the current study was stratified the Ki67 index according to the cut-off point determined by the X-Title software, but other studies have shown different stratification parameters [40]; the precise cut-off value needs to be established with more research for further verification. Furthermore, we did not examine possible mechanistic explanations for the observed association of M-CTC count or Ki67 expression with poor OS. Future studies should elucidate how elevated Ki67 expression and M-CTCs worsen the prognosis of HCC patients.

## Conclusion

Our results suggest that an increase in the number of M-CTCs in preoperative peripheral blood closely correlates with high Ki67 expression in HCC tissues, and that both events promote tumor progression, ultimately leading to poor OS. Our study provides strong evidence that M-CTCs and Ki67 together may serve as a prognostic biomarker for stratifying HCC patients by risk of poor prognosis. These findings may help clinical decision-making and management of HCC patients.

## Supplementary Information

**Additional file 1: Figure S1.** Observation of nuclear Ki67 staining in a case of HCC tissue sample. Ki67-positive staining was identified as the presence of brownish-yellow granules in the nucleus. (A, B) Ki67 ≥ 50%; (C, D) Ki67 < 50%. (A, C) Magnification, 100 ×; (B, D) Magnification, 400 ×. **Figure S2.** Receiver operating characteristic curve analysis of total circulating tumor cell (Total CTC) and mesenchymal CTC (M-CTC) count on prediction of tumor recurrence after curative resection for hepatocellular carcinoma. **Figure S3.** (A), Data represented graphically in a right-triangular grid in which each point (pixel) represents the data from a given set of divisions. The vertical axis represents all possible “high” populations with the size of the high population increasing from top to bottom. Similarly, the horizontal axis represents all possible “low” population with the size of the low population increasing from left to right. (B), The number of patients in each group for a given set of divisions.

**Additional file 2: Table S1.** Capture probe sequences for the *EpCAM, CK8/18/19, vimentin* and Twist genes.

## Data Availability

The datasets used and/or analysed during the current study are available from the corresponding author on reasonable request.

## Abbreviations

AFP: Alpha-fetoprotein
AUC: Area under the curve
BCLC: Barcelona Clinic Liver Cancer
CTC: Circulating tumor cell
CI: Confidence interval
E-CTC: Epithelial circulating tumor cell
E/M-CTC: Epithelial/mesenchymal hybrid circulating tumor cell
EMT: Epithelial-to-mesenchymal transition
HBsAg: Hepatitis B surface antigen
HBV-DNA: Hepatitis B virus DNA
HR: Hazard ratio
HCC: Hepatocellular carcinoma
M-CTC: Mesenchymal circulating tumor cell
MVI: Microvascular invasion
OR: Odds ratio
OS: Overall survival
PBS: Phosphate-buffered saline
PVTT: Portal vein tumor thrombosis
ROC: Receiver operating characteristic
TNM: Tumor / node / metastases
VCE: Vessel carcinoma embolus
