# Supplementary material for: Mesenchymal circulating tumor cells and Ki67: their mutual correlation and prognostic implications in hepatocellular carcinoma
Source: BMC Cancer. 2023 Jan 5;23:10. doi: 10.1186/s12885-023-10503-3 (PMC9814317; doi:10.1186/s12885-023-10503-3)

**Mesenchymal Circulating Tumor Cells and Ki67: Their Mutual Correlation and Prognostic Implications in Hepatocellular Carcinoma**

**Supplementary figures**

**Figure S1.** Observation of nuclear Ki67 staining in a case of HCC tissue sample. Ki67-positive staining was identified as the presence of brownish-yellow granules in the nucleus. (**A, B**) Ki67 ≥ 50%; (**C, D**) Ki67 < 50%. (**A**, **C**) Magnification, 100 ×; (**B**, **D**) Magnification, 400 ×.


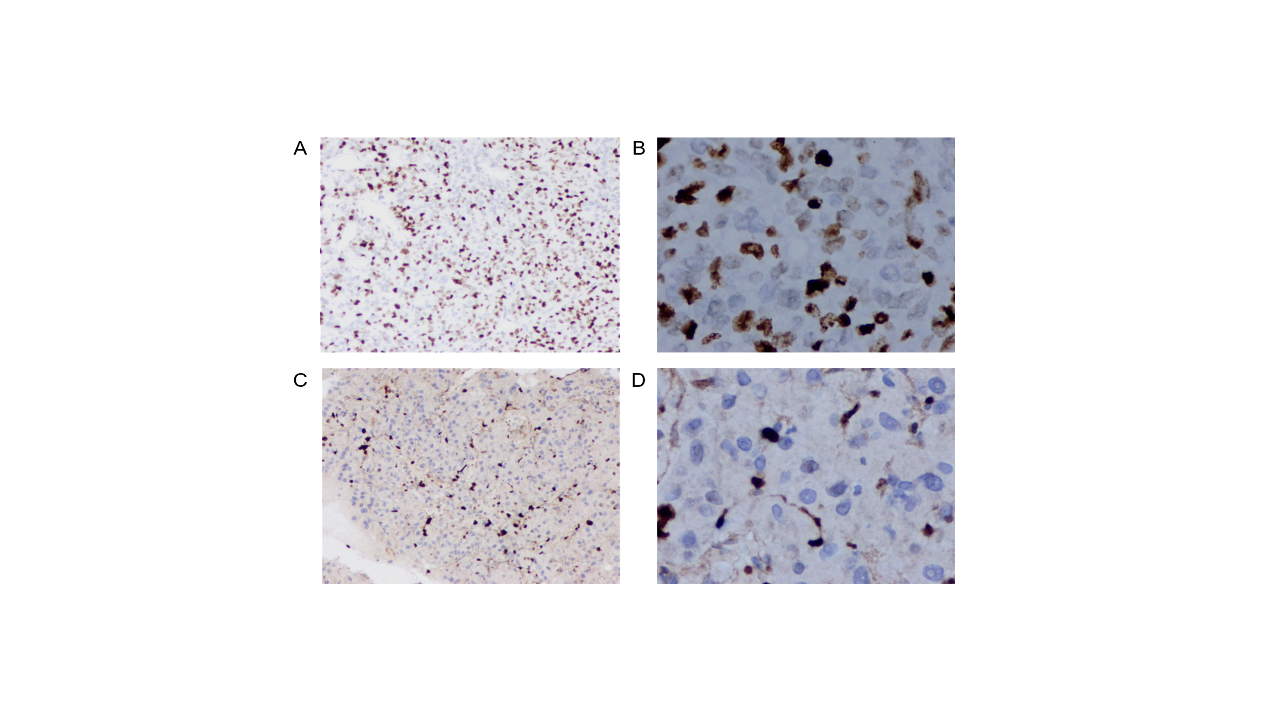


**Figure S2.** Receiver operating characteristic curve analysis of total circulating tumor cell (Total CTC) and mesenchymal CTC (M-CTC) count on prediction of tumor recurrence after curative resection for hepatocellular carcinoma. **Abbreviations**: AUC, area under the curve.


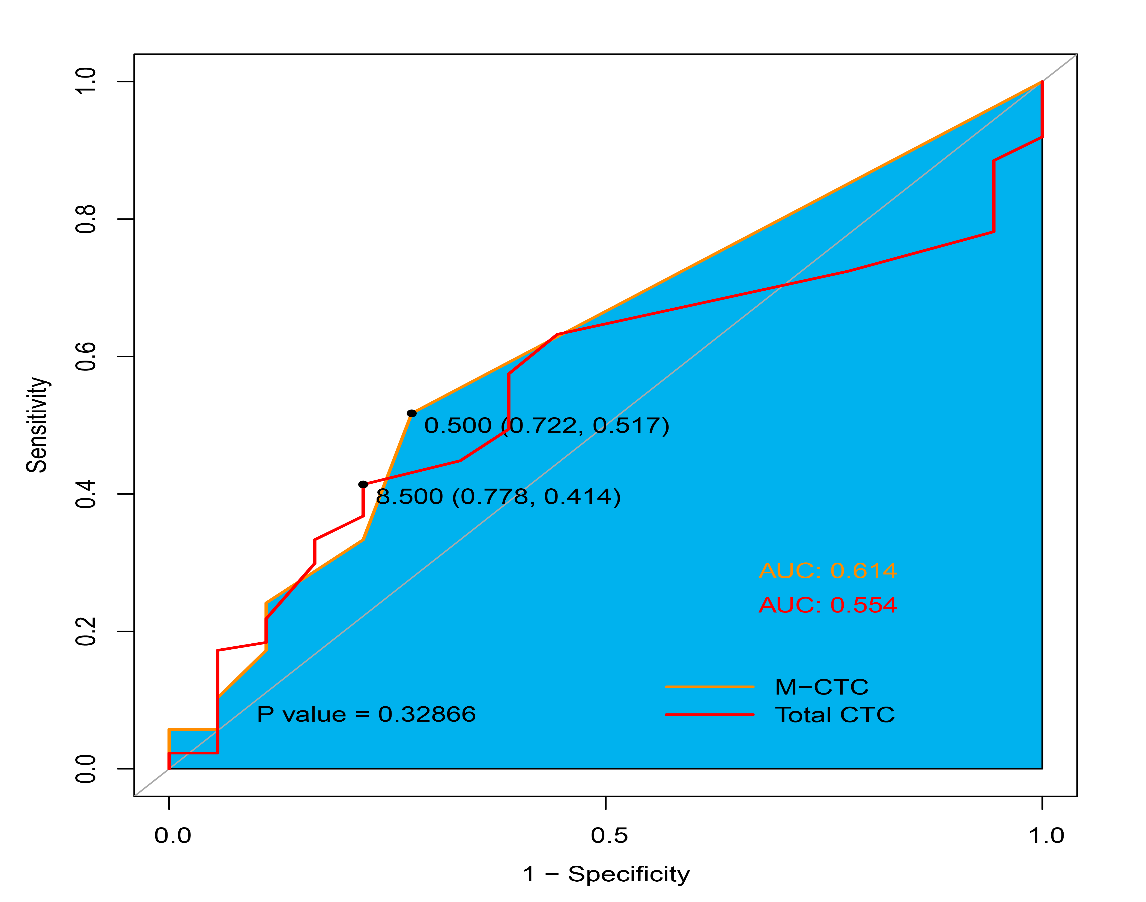


**Figure S3.** (**A**), Data represented graphically in a right-triangular grid in which each point (pixel) represents the data from a given set of divisions. The vertical axis represents all possible “high” populations with the size of the high population increasing from top to bottom. Similarly, the horizontal axis represents all possible “low” population with the size of the low population increasing from left to right. (**B**), The number of patients in each group for a given set of divisions.


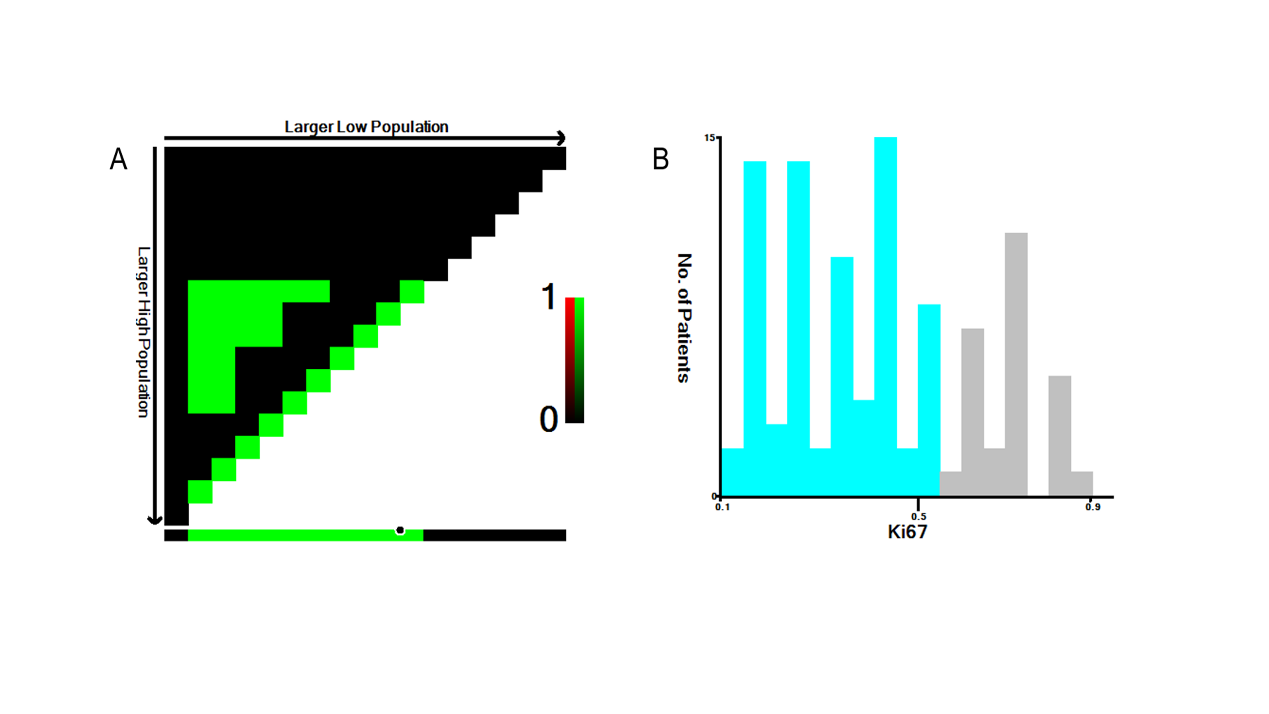

Supplement: Supplementary file 1 — Additional file 1: Figure S1. Observation of nuclear Ki67 staining in a case of HCC tissue sample. Ki67-positive staining was identified as the presence of brownish-yellow granules in the nucleus. (A, B) Ki67 ≥ 50%; (C, D) Ki67 < 50%. (A, C) Magnification, 100 ×; (B, D) Magnification, 400 ×. Figure S2. Receiver operating characteristic curve analysis of total circulating tumor cell (Total CTC) and mesenchymal CTC (M-CTC) count on prediction of tumor recurrence after curative resection for hepatocellular carcinoma. Figure S3. (A), Data represented graphically in a right-triangular grid in which each point (pixel) represents the data from a given set of divisions. The vertical axis represents all possible “high” populations with the size of the high population increasing from top to bottom. Similarly, the horizontal axis represents all possible “low” population with the size of the low population increasing from left to right. (B), The number of patients in each group for a given set of divisions. [file 12885_2023_10503_MOESM1_ESM.docx]
