# Supplementary material for: Mesenchymal circulating tumor cells and Ki67: their mutual correlation and prognostic implications in hepatocellular carcinoma
Source: BMC Cancer. 2023 Jan 5;23:10. doi: 10.1186/s12885-023-10503-3 (PMC9814317; doi:10.1186/s12885-023-10503-3)
Supplement: Supplementary file 2 — Additional file 2: Table S1. Capture probe sequences for the EpCAM, CK8/18/19, vimentin and Twist genes. [file 12885_2023_10503_MOESM2_ESM.docx]

**Table S1.** Capture probe sequences for the *EpCAM, CK8/18/19, vimentin* and *Twist* genes.

| Gene name | Sequences (5´-3´) |
| --- | --- |
| EpCAM | 5´-TGGTGCTCGTTGATGAGTCA  AGCCAGCTTTGAGCAAATGA-3´ |
| CK8 | 5´-CGTACCTTGTCTATGAAGGA |
|  | ACTTGGTCTCCAGCATCTTG-3´ |
| CK18 | 5´-AGAAAGGACAGGACTCAGGC |
|  | GAGTGGTGAAGCTCATGCTG-3´ |
| CK19 | 5´-CTGTAGGAAGTCATGGCGAG |
|  | AAGTCATCTGCAGCCAGACG-3´ |
| Vimentin | 5´-GAGCGAGAGTGGCAGAGGAC |
|  | CTTTGTCGTTGGTTAGCTGG-3´ |
| Twist | 5´-ACAATGACATCTAGGTCTCC |
|  | CTGGTAGAGGAAGTCGATGT-3´ |
